# Understanding the success factors of MOOCs’ retention intention: A Necessary Condition Analysis

**DOI:** 10.1371/journal.pone.0310006

**Published:** 2024-11-07

**Authors:** Zhang Huacui, Md. Nahin Hossain, Kong Zhen, Nilesh Kumar

**Affiliations:** 1 School of Music, Shandong Women’s University, Jinan, China; 2 Department of Business and Technology Management, Islamic University of Technology- IUT, OIC, Dhaka, Bangladesh; 3 Business School, Shandong Jianzhu University, Jinan, China; 4 School of Psychology, Zhejiang Normal University, Jinhua, China; 5 School of Business Administration, Zhejiang Gongshang University, Hangzhou, China; Nanyang Technological University, SINGAPORE

## Abstract

This paper seeks to explore the influence of success factors, specifically motivation and course quality, on MOOC retention intention. Going beyond a mere examination of these motivational and quality factors, the study investigates students’ motivation, considering needs, interests, course system, content, and service quality. Methodologically, a questionnaire survey was conducted, collecting data from 311 students enrolled in online courses. To ascertain the impact of interest or need-based motivation on students’ retention rates, a Structural Equation Model (SEM) was employed. Subsequently, Necessary Condition Analysis (NCA) was utilized to identify the essential factors and components in each area. SEM results revealed a positive influence of motivational factors and quality issues on students’ behavior. Retention behavior was notably affected by academic and professional needs, along with personal interests. Furthermore, course content and service quality demonstrated a significant effect on students’ perseverance behavior. NCA results identified academic motivation and system quality as having a substantial impact on retention behavior, while personal motivation and technological motivation had a comparatively smaller effect. Practically, the findings suggest that course developers should consider students’ academic and personal requirements when designing online courses. Additionally, providing students with the ability to customize course and system content according to their needs is crucial. Timely problem-solving attitudes from service providers are essential for ensuring student retention.

## 1. Introduction

Massive Open Online Courses (MOOCs) represent a resourceful platform for individuals to engage in skill-based knowledge or open-access courses via the internet, offering opportunities to absorb new insights, acquire skill sets, and broaden occupational horizons. However, a recent trend of increased dropout rates in MOOCs has raised concerns among education researchers [1], urging scholars to examine the efficacy of these platforms [1]. Numerous studies have been conducted to identify the causes of dropout and develop strategies to mitigate the dropout percentage [2–4]. In particular, scholars emphasize that motivation emerges as a fundamental factor influencing learners’ retention in MOOCs [2–4].

According to Maya-Jariego, Holgado [5], learners can be categorized into three groups based on their internal motives, external motives, and intention of persistence, highlighting a significant connection between motivation intensity and MOOC retention and satisfaction. Several scholars have emphasized the fundamental role of motivation in interpreting learners’ behavior, asserting that internal motivation significantly influences the intention to continue with MOOCs. Tang and Chaw [6] validated these findings, establishing a direct link between participants’ motivation and course completion in MOOCs. Furthermore, a systematic review identified quality issues, such as unclear course design, lack of interaction, learner experience, time management, and mastery of MOOCs course language, as factors influencing persistence in MOOC curriculums Goopio and Cheung [3].

Earlier research focused on quality factors motivating students to sustain engagement with specific MOOC platforms [7]. Given the divergent quality observed among MOOC platforms, it becomes crucial to comprehend the essential quality and motivational factors influencing students’ retention intentions in MOOCs. This understanding is critical for MOOC developers to design appealing content, attract more students, and assist educators and vendors in formulating strategies to enhance MOOC utilization.

Significantly, there has been a noteworthy resurgence of interest in MOOCs during the COVID-19 pandemic, following the initial enthusiasm in 2012 [8]. The pandemic-induced quarantine measures in many countries have heightened interest in MOOCs, as learners actively seek open access to educational material anytime and anywhere. In 2020, the percentage of participants registering on these platforms accounted for one-third of all participants ever recorded, excluding China [9]. Noteworthy statistics indicate a substantial increase, with participants surging from 2 million in 2012 to 180 million in 2020, courses escalating from 250 to 16.3K, and university partnerships for platforms rising from 40 in 2012 to 950 in 2020. Additionally, there has been a surge in publications on MOOCs in recent years [10, 11].

While prior research on MOOCs has explored learners’ motivation [12, 13], behaviour [14], and dropout rates [15, 16], limited attention has been given to investigating the success factors crucial for reducing dropout rates and enhancing motivation and quality in MOOCs. Identifying the factors that contribute to retaining learners helps in designing courses that are engaging and conducive to learning. This includes understanding which quality aspects of course content, structure, or presentation keep learners motivated and committed. Higher retention rates often correlate with better learning outcomes [17]. When learners stay engaged and complete courses, they are more likely to achieve the intended learning objectives, leading to improved skill development. Additionally, continuously monitoring these success factors allows course providers to iteratively improve the necessary conditions for MOOCs. This involves gathering feedback from learners, analysing data on completion rates, and adjusting course elements based on empirical evidence of what works best [18]. The term ’Necessary Condition’ might be potentially misleading, as the mere existence of a factor does not automatically ensure success [19]. Necessary Conditions, akin to Herzberg [20] hygiene factors, do not guarantee contentment but are indispensable to prevent discontent. Similarly, success factors are essential to improving dropout rates, and their absence has a direct negative impact on other factors. Researchers [21] recommend investigating necessary conditions that have no alternatives for improving dropout rates. Simply having all motivational and quality factors present does not guarantee a high retention rate. Hence, this study aims to address:

Q1: Are motivational factors (Need-Based or Interest-Based) essential for reducing dropouts?
Q2: What are the essential quality factors (System, Information, Service, etc.) to improve retention intentions?

## 2. Literature review of success factors

### 2.1. Learners’ motivation

Motivation consistently plays a crucial role in sustaining learners’ engagement with online courses. This learner-centric motivation typically takes two distinct forms: a) Need-based motivation, and b) Interest-based motivation. The primary objective of need-based motivation in registering for or completing a MOOC is to address gaps in an individual’s knowledge, skills, or attitudes. Within the realm of need-based motivation, learners actively participate in educational pursuits to fulfill perceived deficiencies [22]. Theoretical frameworks grounded in need-based perspectives posit that while external factors contribute to behavioral motivation, intrinsic motivational factors exert a more significant influence.

#### i) Need-based motivations encompass academic, course, and professional motives

Recognizing need-based motivations in academic, course, and professional settings highlights the importance of personal growth. Students and professionals are often driven by the need for knowledge, skill development, and career advancement [23]. Academic motives entail students’ aspirations regarding academic subjects, suggesting that those with heightened academic motivation tend to achieve greater academic success [24]. Conversely, low academic motivation correlates with increased academic failure [25]. A review of MOOC-related literature supports the influence of factors for instance academic self-efficacy [26, 27], teaching presence, innovative teaching tools [17], knowledge acquisition, certification attainment, and credit accrual on MOOC retention [28, 29].

Course motives pertain to the impact of course structure, design, and content on attracting learners while deciding on MOOC enrollment and completion. This perspective aligns with goal-setting theories, suggesting that individuals are motivated to fulfill specific needs [30]. For instance, students may be motivated by the need to achieve good grades (academic), to understand course content deeply (course), or to gain skills relevant to their future careers (professional) [31]. Research indicates that factors such as the complication and struggle of course content, course timing, perceived course effectiveness, and course design significantly influence participants’ decisions to continue or drop out of MOOCs [32].

Professional motives underscore the importance of acquiring new knowledge and skills for occupational development [18]. This motive highlight MOOCs as vital for acquiring new knowledge and skills, essential for occupational development. They offer flexible, accessible learning, enabling individuals to stay competitive, upskill, and adapt to changing job markets, enhancing career prospects and fostering continuous professional growth [33]. Thus, participants are motivated by the potential benefit of using MOOCs for their development and career advancement [22, 34]. Existing literature affirms that the need for professional development serves as a significant motivator for MOOC users. Workplace-related factors, course relevance to job, and economic mobility also impact learners’ MOOC retention [9].

#### ii) Interest-based motivation includes personal, social, and technological motives

Interest emerges as a potent motivator for MOOC enrollment and learning (Tsai et al., 2018). Interest-based motivation delineates that interest serves as a major motivational source as individuals interact with their environment. Research indicates that interest can be categorized into personal and situational interests [35]. Personal motives, driven by family circumstances, curiosity, personal growth, enjoyment, personal interest, and prior experiences, significantly influence participants’ decisions to drop out or complete MOOCs [36].

Social motives encompass the human need for connection and acceptance by others. Social factors such as social presence, support, interactions, meeting new people, friends taking a course, and connection with others impact MOOC users’ decisions to choose and complete a course [1, 36].

Studies on MOOCs have explored the role of technological factors in motivating learners to actively participate and successfully complete courses. The research has emphasized the significance of various technological aspects in preventing dropout. Notably, perceived ease of technology use [37–39], media richness [40], potential for interactivity [40, 41], visual design, infrastructure limitations [42], accessibility challenges [41, 43, 44], and the convenience of technology have been identified as influential factors affecting participants’ engagement with MOOCs. These factors not only impact the use of MOOCs but also influence learners’ decisions regarding dropout or their intention to continue using MOOCs.

In recent years, Massive Open Online Courses (MOOCs) have garnered significant attention as an innovative model for technology-enhanced learning in higher education. These courses offer a multitude of educational opportunities by providing a large number of learners with access to free online course worldwide. Despite this, challenges such as low completion rates and pedagogical issues related to assessment and feedback have raised concerns, suggesting a potential tradeoff between scale and efficacy. The sustainability of the MOOC platform hinges on the continuous participation of learners, a crucial metric for assessing system success [45, 46].

### 2.2. Quality of MOOCs

In the MOOC context, the definitions of system quality, course quality and service quality differ from those in general information systems. Previous research [47] identified components of system quality in a course content management system, including reliability, flexibility, integration, accessibility, and timelines [48]. Nevertheless, following research posits that the comparative prominence of every factor of system quality varies.

System quality, in the MOOC platform, is conceived as the integration of system functions and the reliability of system operation as perceived by users [49]. When users believe that the MOOC platform delivers a fully functional learning system, their intention to continue participating in MOOCs is positively influenced. Lu and Dzikria [50] illustrated the positive impact of system quality on users’ intentions using the internet as an example. Despite the internet’s popularity, various system quality factors can lead to the discontinuation of its use [33]. Yang, Shao [7] asserted that system quality is a key driver of consumer perception and subsequent online behavior.

Information quality, a joint determination of the source and content of information [51], is crucial in the MOOC context. Users’ perception of course quality is influenced by factors such as the lecturer’s knowledge, the authority of the course content, and the lecturer’s teaching attitudes. In this study, course quality replaces information quality and is defined by knowledgeability, authority of course content, and lecturers’ teaching attitudes. When users perceive online course as possessing high knowledgeability and authority, with teachers demonstrating a serious and well-prepared approach to teaching, their intrinsic motivation to continue studying is fully stimulated. Course quality has been shown to significantly impact users’ use of information systems, particularly in the context of e-learning systems [52]. Bhattacherjee, Perols [51] argued that course quality positively influences users’ online behaviors, and Liu and Pu [48] found that it enhances the behavioral intention to reuse e-learning systems.

Service quality is defined as a global judgment or attitude relating to the superiority of a service [53]. Within the scope of this study, service quality pertains to the comprehensive support provide by the MOOC’s service provider, encompassing assistance extended to learners on the MOOC platform. This support includes offering professional guidance, aiding users in practical exercises, assigning homework, and organizing examinations. According to several scholars, service quality is a key predictor of repetitive behavior intentions. A user’s perception of high service quality increases the likelihood of continued use of the information system in the future. Researchers Ding and Shen [35]confirmed a positive correlation between service quality and continuance intentions. Moreover, several studies have suggested that users, perceiving perfect service from and information system, develop a strong sense of satisfaction and intend to reuse it in the future [54–58].

In light of the aforementioned comprehensive literature review and articulated arguments, this study endeavors to undertake a Necessary Condition Analysis. The primary objective is to clarify the fundamental factors pertaining to motivation and quality that are indispensable for enhancing the retention rate.

## 3. Research methodology

### 3.1. Sample

This study selected leading MOOC platforms to achieve its research objectives. Approximately 1,500 courses are available on these platforms in Bangladesh. Therefore, for statistical analysis, this study focused on participants with MOOC experience in Bangladesh. It utilized a combination of stratified random sampling and convenience sampling methodologies. Stratified random sampling was employed to ensure comprehensive data collection from all major cities within each division of Bangladesh [9]. Concurrently, convenience sampling specifically targeted respondents who used MOOCs, identified through an initial screening question in the online survey. The study collected 294 complete responses between July 1, 2022, and October 15, 2022, excluding 31 incomplete responses (refer to Table 1 for demographic information of the respondents). Additionally, semi-structured questionnaire surveys were distributed to the participants, and a group of five interviewers gathered information from the participants after addressing missing values.

**Table 1:** Demographic profile.

Items	Types	Number	Percentage
Gender	Male	160	54%
	Female	134	46%
Age	Below 18	29	10%
	18–30	97	33%
	Above 30	168	57%
Education	High School	29	9%
	Bachelor	122	41%
	Masters and above	143	50%

Questionnaire was classified into two sections. Section-1 contained 27 questions on variables considered in the following research. Scales were adopted from existing literature with minor context specific adjustment (See–S1 Appendix). Section-B consisted demographic profile for example gender, age, and education.

### 3.2. Ethical approval and consent to participate

This study was approved by the ethics committee of Zhejiang Gongshang University, and data collection involving human participants adhered to the principles outlined in the Declaration of Helsinki (202111/IRB/14). The procedure for obtaining consent to participate was approved by the ethics committee overseeing this study. All participants were informed about the research objectives and provided virtual informed consent before answering the main research questions, ensuring the privacy of their data. Public sharing of data would violate the compliance protocols approved by the research ethics board. However, interested parties can request access to the data by contacting the ethics committee of Zhejiang Gongshang University at 2021160@mail.zjgsu.edu.cn, providing a valid reason for their request.

### 3.3. Measures

**Fig 1:**
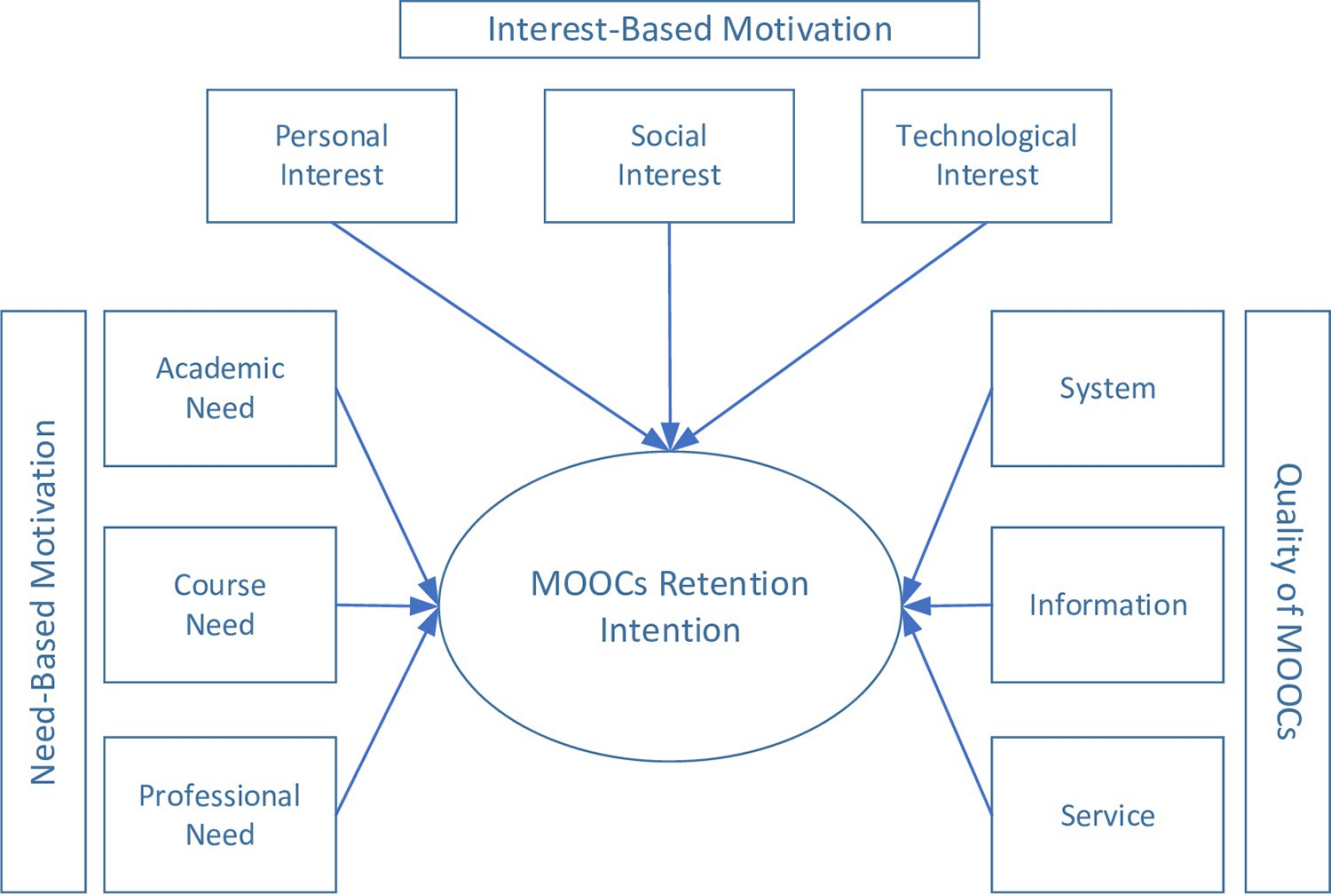
Research model.

Literature has classified following success factors that have presented in Fig 1 (Research Model): 1): Academic motives, 2): Course motives, 3): Professional motives, 4): Personal motives, 5): Social motives, 6): Technological motives, 7): System quality, 8): Information quality, and 9): Service quality. In order to avoid idiosyncratic variance and enhance construct validity, all items were clarified to participants, verbally and in writing. All outcomes were later discussed with participants, enabling us to identify whether measured variables represented the intended case and factors. Followed by Finstad’s [59] instructions, we measured single-item questions on 7-point Likert scale (1 = “No presence,” to 7 = “Full presence”) to avoid measurement error.

### 3.4. Data analysis

Available literature on motivation and quality’s role in learners’ continuance behavior emphases on the net effects, signifying conventional approaches i.e., SEM or multiple regression analysis [60–62]. Yet, these techniques do not evaluate casual complexities [63]. As a result, the present study did not only evaluate the net effects of individual components of motivation and quality for diagnosing retention behavior using SEM, but it also investigated significance level of each component of motivation and quality using NCA that are required for successful completion of online courses. The 9 components, necessary for students to reduce the dropout rate, are as follows, underlying 6 for motivation (Academic motives, Course motives, Professional motives, Personal motives, social motives, Technological motives,) and 3 for quality (system quality, information quality, service quality).

This study employs R programming language as its primary analytical tool to investigate. R was chosen for its robust statistical capabilities and flexibility in handling complex data sets, aligning with the study’s quantitative approach.

### 3.5. Common method bias

Variance Inflation Factor (VIF) is calculated to measure Common Method Bias (CMB). All the factors need to be within the range of ’+/- 3.’ As shown in the VIF column of Table 2, all the factors fall within this range. Therefore, CMB is not present in the study.

**Table 2:** Construct reliability and validity.

	Cronbach’s alpha	Composite reliability (rho_a)	Variance Inflated Factor (VIF)	Average variance extracted (AVE)
Academic Needs	0.857	0.863	4.136	0.701
Course Needs	0.865	0.869	2.903	0.711
Information Quality	0.860	0.866	3.277	0.704
MOOCs Retention	0.880	0.881	-	0.807
Professional Needs	0.768	0.768	2.925	0.683
Personal Motives	0.853	0.854	2.515	0.694
Service Quality	0.857	0.864	2.863	0.699
Social Motivation	0.788	0.737	2.674	0.618
System Quality	0.826	0.835	2.734	0.655
Technological Motivation	0.830	0.837	4.136	0.746

## 4. Analysis and findings

Considering the psychometric properties, including construct reliability and validity, of all variables (see Table 2) and discriminant validity (see Table 3) prior to regression analysis, numerical values demonstrate statistical support, meeting the threshold criteria. In particular, variance inflation factor (VIF) indicates no multicollinearity, no method bias and average variance extracted (AVE) justifies convergent validity. Overall, data analysis and categorization disclosed that academic, professional, personal, service quality, system quality and information quality have statistically significant correlation on retention rates. In contrast, social motivation along with technological and course motivations are not significant. Additionally, findings demonstrate that components of Needs-based (academic, professional), Interest-based (personal), and Quality (service, system, information) of MOOCs are regressed on leaners’ retention behavior.

**Table 3:** Discriminant validity (HTMT ratio).

	(AM)	(CQ)	(IQ)	(MR)	(PrfM)	(PerM)	(SrvQ)	(SM)	(SQ)	(TM)
Academic Motives (AM)										
Course Motives (CQ)	0.851									
Information Quality (IQ)	0.670	0.832								
MOOCs Retention (MR)	0.653	0.697	0.871							
Professional Motivation (PrfM)	0.630	0.600	0.646	0.844						
Personal Motives (PerM)	0.762	0.662	0.762	0.625	0.839					
Service Quality (Srv Q)	0.774	0.673	0.778	0.576	0.547	0.851				
Social Motivation (SM)	0.660	0.546	0.682	0.602	0.652	0.679	0.876			
System Quality (SQ)	0.552	0.539	0.640	0.608	0.682	0.553	0.607	0.807		
Technological Motivation (TM)	0.662	0.654	0.500	0.559	0.687	0.650	0.662	0.676	0.713	

### 4.1. Necessary Condition Analysis

Necessary Condition Analysis scrutinizes the necessity of a condition. As defined by Dul et al. [64], “*a necessary cause is a constraint*, *a barrier*, *an obstacle*, *a bottleneck that must be managed to allow the desired outcome to exist*. *Every single necessary cause must be in place*, *as there is no additive causality that can compensate for the absence of the necessary cause*.*”* Precisely, the necessary condition is required to be met for the manifestation of expected results, yet doesn’t assure the expected results [19]. Such that, it is grounded on multiplicative causality (i.e., Y 5 a. b1X1. b2X2. b3X3…) where zero value of any cause would diminish the results to zero. According to Garg [65], the necessary condition shall be assessed using scatter diagram which considers gratitude on X-axis and teacher leadership on Y-axis. Then, a ceiling line which separates empty zone (zone without any observation) from the full zone with observations is drawn. Garg further added that the existence of empty upper-left corner region of the graph ratifies the necessity of the condition. Effect size measures strength or degree of necessity of the condition. Value of effect size lies between zero and one such that:

0 < d < 0:1 = small effect size;

0:1≤d < 0:3 = medium effect size;

0:3≤ d < 0:5 = large effect and

0:5≤ d <1:0 = very large effect size:

Conclusively, threshold limit of 0.1 is indispensable to draw categorical proof, reflecting the existence of necessity of predictor variable [65]. Therefore, the present study analyzed data using regression and necessary condition analysis for examining necessity of motivation and quality for understanding critical success factors of learners’ retention intention.

### 4.2. Need-based motivation

#### Academic motivations and MOOCs retention rate

The effect of academic motivation is fundamental to the learner’s success, endorsing that students with high motivation tend to accomplish more than students with low academic motivation [25]. Previous literature has also supported those academic motives, for example student’s self-efficacy [38, 40], teaching presence, applying innovation teaching tools (i.e., flipped classroom and query-based learning), and learning new skills, effect students’ retention.

**Fig 2:**
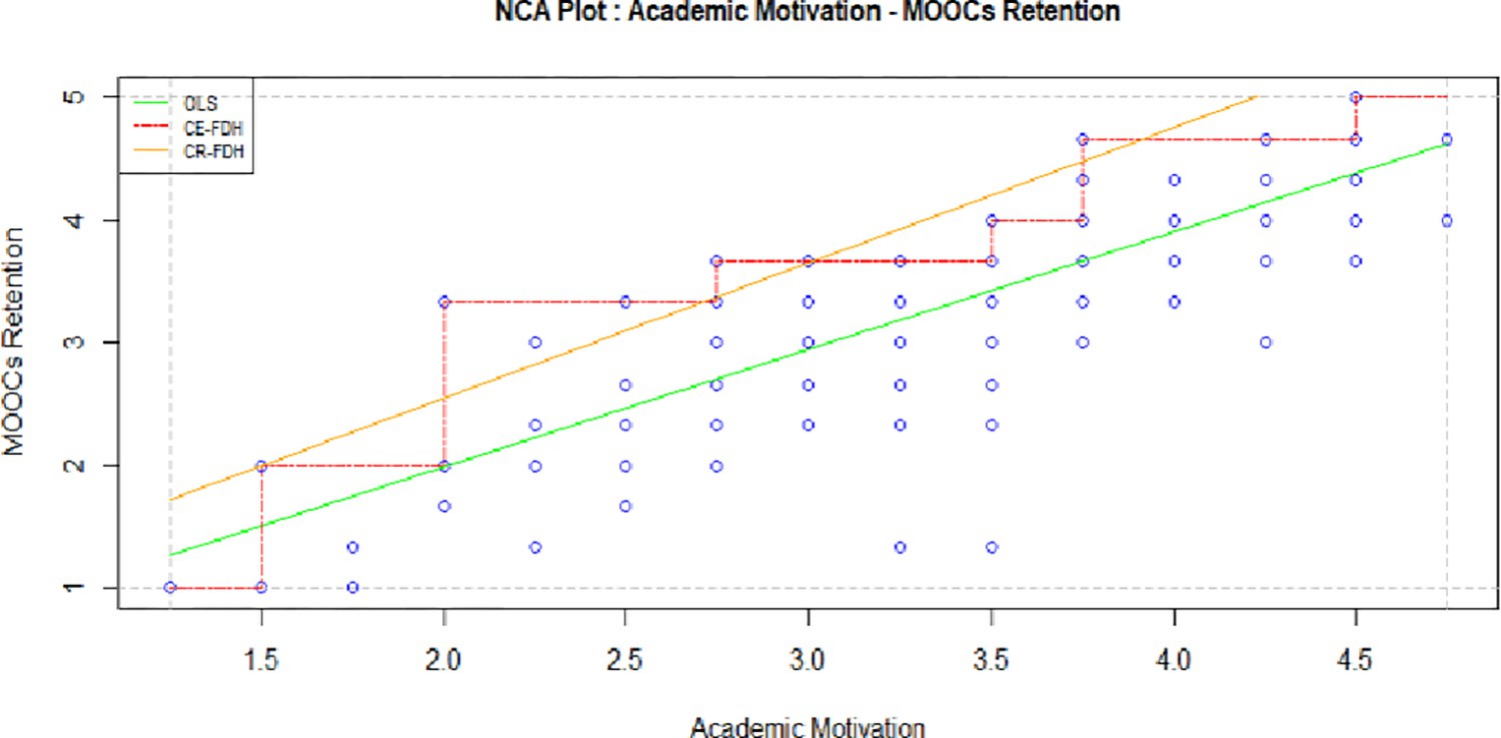
NCA of academic motivation and MOOCs retention.

Table 4 provides regression analysis outcomes, exhibiting academic motivation as the independent construct. The test found a statistically significant standardized regression coefficient (β 0.347) and supported academic motivation’s impact on learning retention intention (t value = 7.010, p = 0.000). Table 5, indicating NCA, confirmed significant effect (CE-FDH 0.376) by reporting empty upper-left-zone of the curve in Fig 2. Here academic motivation has large effect on MOOCs retention. It is observed, only (1.5–2) 0.5 changes in academic intention increases 3.76 levels of retention behavior in 5 rating scale. This advocates the necessity of academic motivations to retain learners. Thus, it is concluded to be necessary condition for this first dimension of motivation.

**Table 4:** Regression result (Sufficiency test).

	Unstandardized coefficients	Standardized coefficients	T value	P value	2.5%	97.5%
Social Motivation	0.020	0.020	0.487	0.626	-0.061	0.101
Academic Motives	0.347	0.347	7.010	0.000	0.250	0.444
Technological Motivation	0.033	0.033	0.815	0.416	-0.046	0.112
Professional Motives	0.365	0.365	8.766	0.000	0.283	0.447
Service Quality	0.166	0.166	4.288	0.000	0.090	0.242
Personal Motivation	0.071	0.071	2.056	0.041	0.138	0.003
Course Motives	0.042	0.042	1.009	0.314	-0.040	0.123
System Quality	0.238	0.238	5.983	0.000	0.316	0.160
Information Quality	0.295	0.295	6.701	0.000	0.209	0.382

**Table 5:** Effect size (Necessity analysis).

*Need-based Motivation*	CE-FDH	CR-FDH	Effect
Academic Motives–MOOCs Retention	0.376	0.357	large
Course Motives—MOOCs Retention	0.179	0.234	medium
Professional Motivation—MOOCs Retention	0.203	0.248	medium
*Interest-based Motivation*			
Personal Motives—MOOCs Retention	0.308	0.270	large
Social Motivation—MOOCs Retention	0.037	0.019	small
Technological Motivation—MOOCs Retention	0.312	0.237	large
*Quality of MOOCs*			
Service Quality—MOOCs Retention	0.265	0.273	medium
System Quality—MOOCs Retention	0.327	0.327	large
Information Quality—MOOCs Retention	0.323	0.314	large

#### Course motivation and MOOCs retention intention

**Fig 3:**
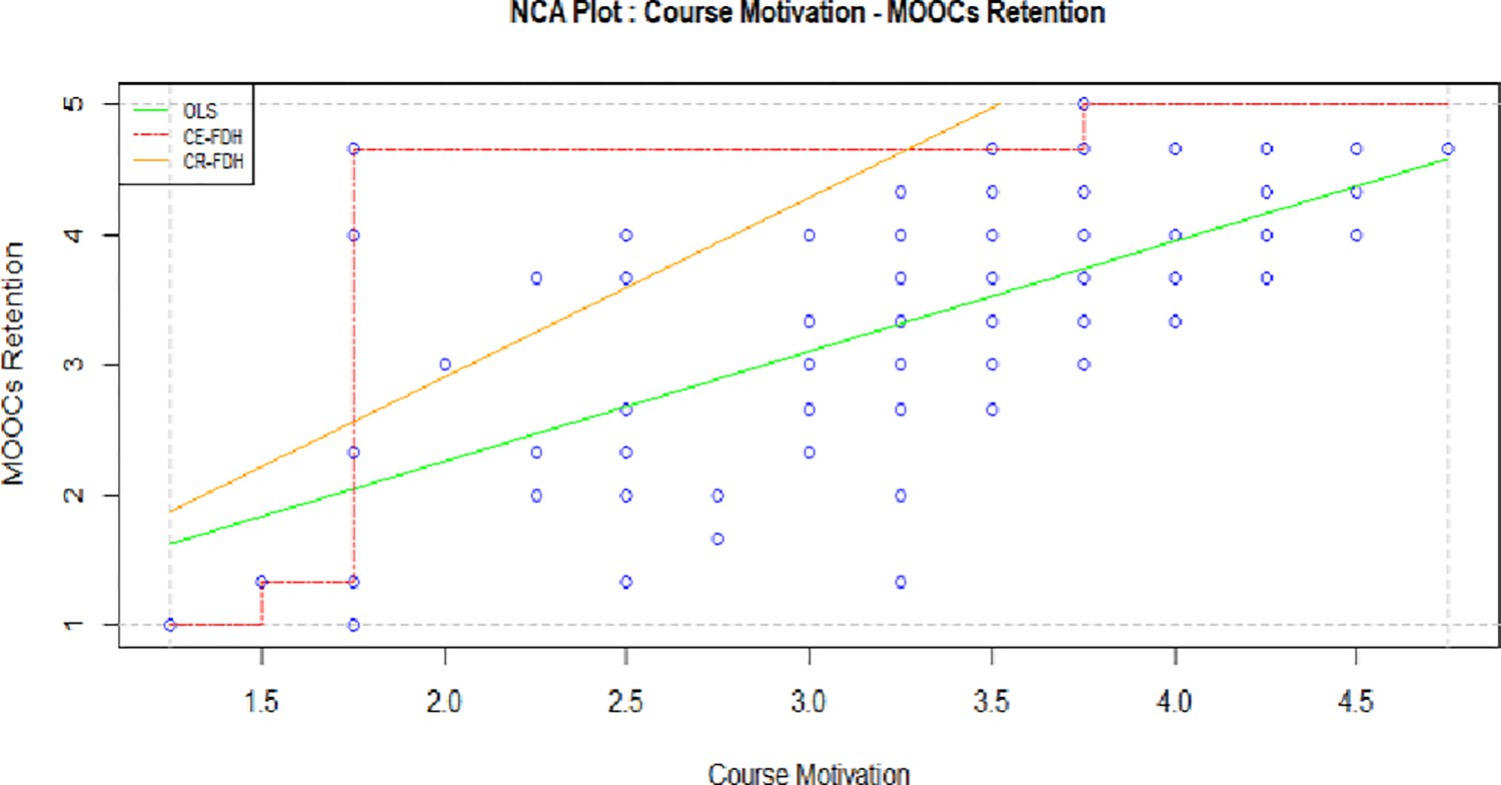
NCA of course motivation and MOOCs retention.

This motivation denotes to the potential of course structure, design, and content to draw participants’ interest to decide whether to enroll and finish MOOCs or not. In particular, Chika-James [66] disclosed that the complexity and difficulty of course content impacts participants’ dropout in MOOCs. More specifically, participants look for course content that is effective and easy to follow. However, this study found that (see Table 4), course motivation has zero (P value-0) or no impact on students’ retention behavior. This result is inconsistent with the NCA. In Table 5 and Fig 3 report that course motivation defines medium effect size (CE-FDH 1.79) in learners’ retention intention. The findings suggest that the quality of course structure, design and content is imperative, thus validating it as necessary component to retain students. Additionally, course motivation is vital to complete the first stage, yet requiring other supporting factors to reach at sufficient level.

#### Professional motives

**Fig 4:**
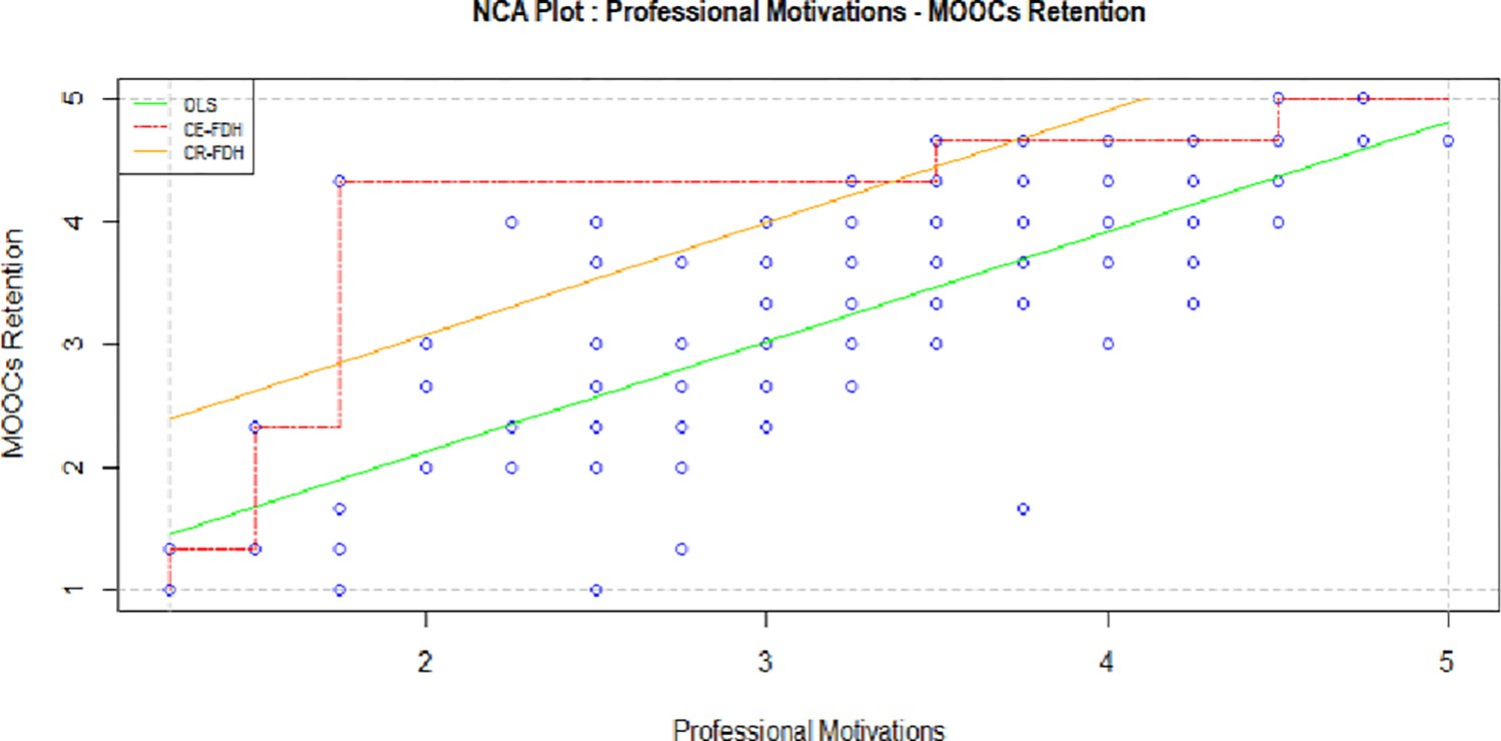
Professional motivations and MOOCs retention.

MOOC is an opportunity for absorbing and acquiring new insights, skillsets, and expanding occupational horizons [67]. Professional motives refer to professional development and career reasons why participants use MOOCs and intend to complete them. Previous studies posited that career-related needs are key players and are favorably motivating for MOOCs users [68–70]. In particular, scholars narrated that course relevancy with one’s job has positive association with user’s intensity of learning and satisfaction [71]. Statistically, Table 4 and Fig 4 exhibit significant support for previous findings. Although, according to regression analysis and hypothesis test (β 0.365, P value- 0), professional motive is sufficient. However, necessary conditions identified professional motives’ medium effect (CE- FDH 0.208) on perseverance human intention. Professional motives such as; work circumstances [60, 72], workplace knowledge and experience [36, 73] may not always necessarily factors to motivate the learners’ retention behavior. Other supporting factors like monetary benefits, personal growth together is sufficient for fulfilling professional motivation [74]. Thus, NCA showed professional motivation is not salient factors for MOOCs retention behavior.

### 4.3. Interest-based motivation

#### Personal motives

Personal motives signify one’s personal reasons for why one gets enrolled on MOOC. According to previous findings, following reasons could be motivating and demotivating criteria for participant to dropout or complete MOOCs; 1): family circumstances [60], 2): curiosity [75], 3): personal growth [76], 4): enjoyment and boredom [77], 5): personal interest [75], 6): personal reasons [36], and 7): prior experiences [73].

**Fig 5:**
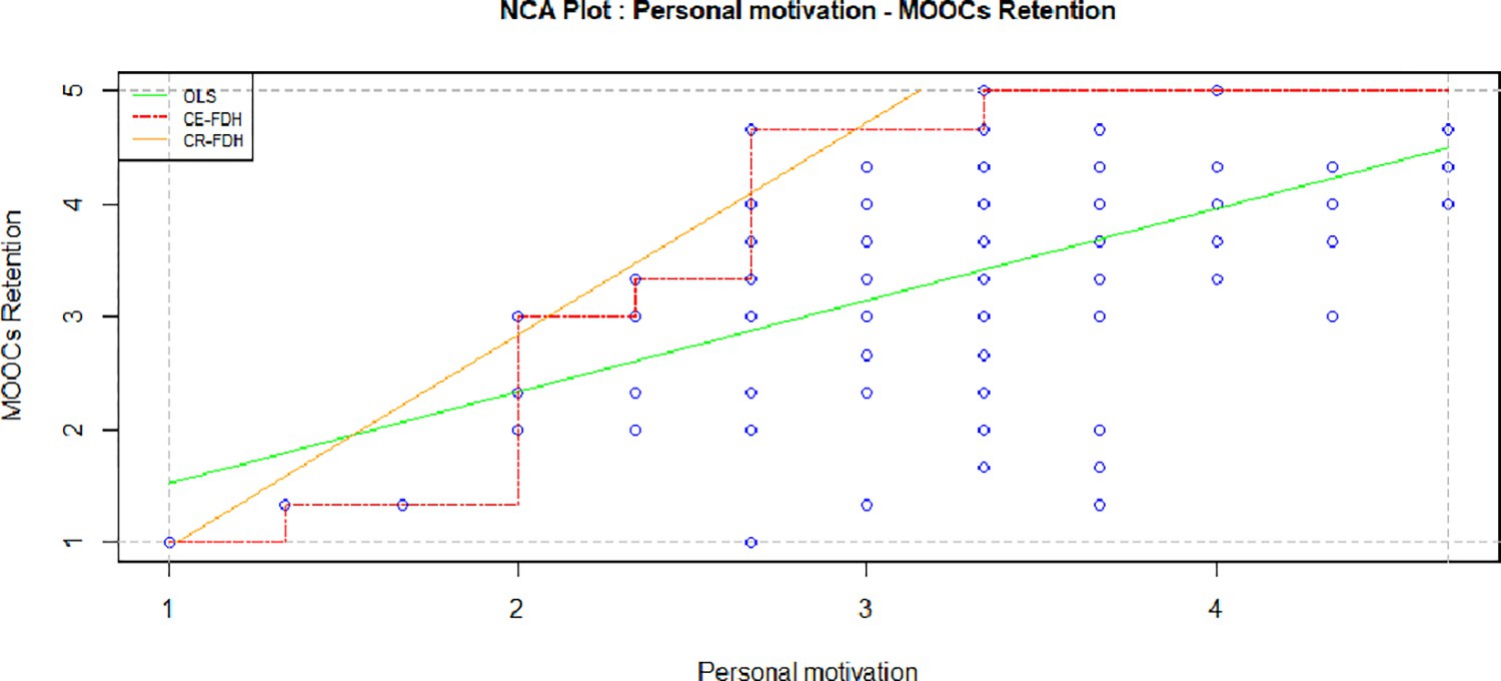
Personal motives and MOOCs retention.

Considering the low impacts of certifications in MOOCs retention [45], having personal motives to take a MOOCs tend to play a major role in MOOCs completion [78]. However, according to Necessary condition analysis, personal motive is sufficient condition, yet not necessary. Table 4 and Fig 5 show that personal drive can be satisfied by other factors and have few or no effect on retention behavior.

#### Social motives

**Fig 6:**
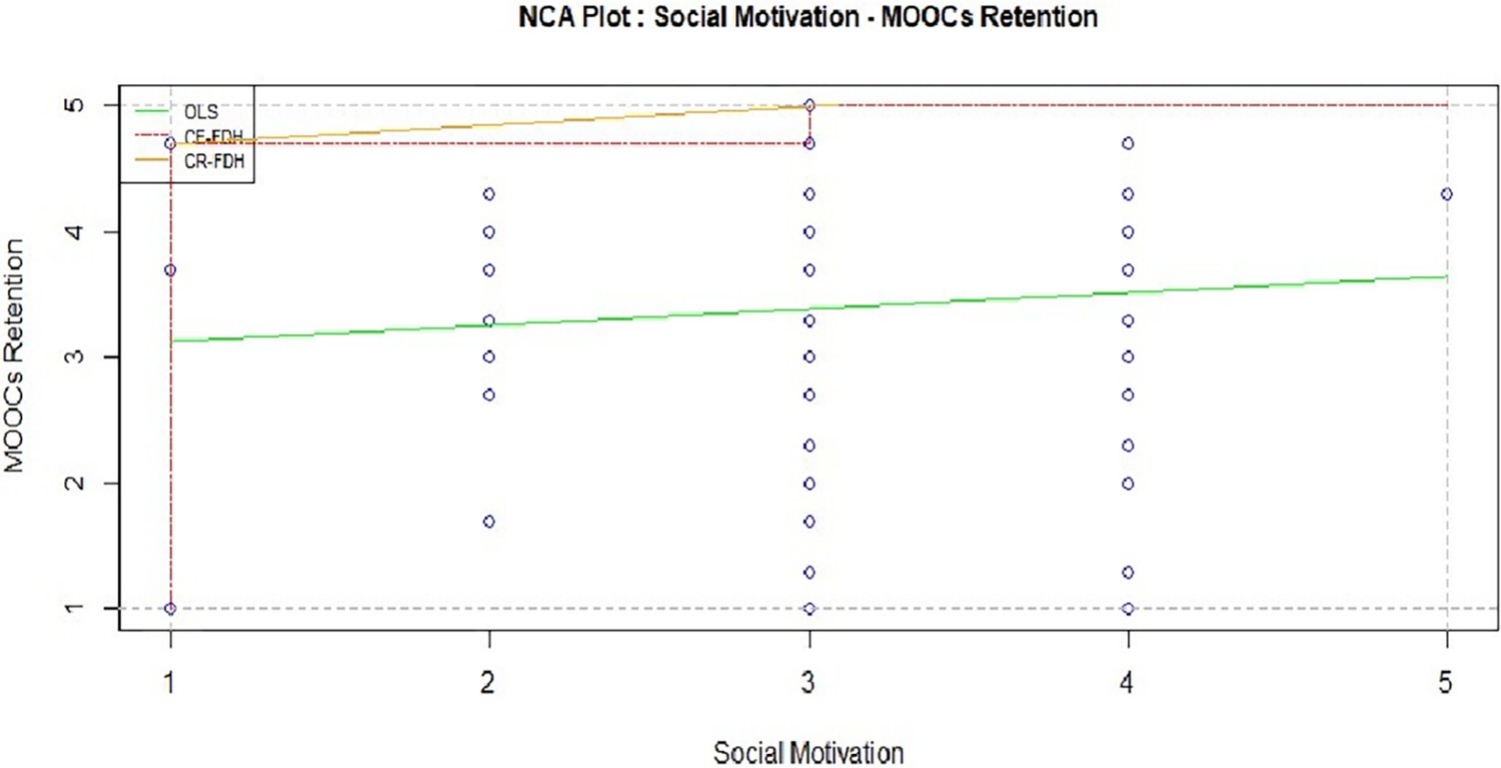
Social motives and MOOCs retention.

Social motivations in MOOCs make the course more attractive for learners and improve the level of participation, engagement, performance, and attitude toward using MOOCs–signifying important factors in MOOCs completion [1, 72, 77, 79]. However, in the case of continuous use of Massive Open Online Courses (MOOCs), it is not solely driven by social motives. While some individuals may be influenced by the desire to learn alongside peers or gain social recognition, the decision to enroll in MOOCs is typically multifaceted. Educational and skill development, flexibility, career advancement, cost-effectiveness, personal interest, and a preference for self-directed learning are among the diverse motivations [6, 80]. MOOCs cater to a wide range of individual needs, and the appeal lies in the autonomy they offer, making them accessible for various purposes beyond social considerations. The statistical findings are also similar with the above discussion. In Tables 4 and 5, it is observed that social motivation is neither sufficient (Standardized coefficients 0.020, P- value 0) nor necessary condition, as depicted in Fig 6 (CE-FDH: 0.037, small effect size), for students’ retention behavior.

#### Technological motivation

**Fig 7:**
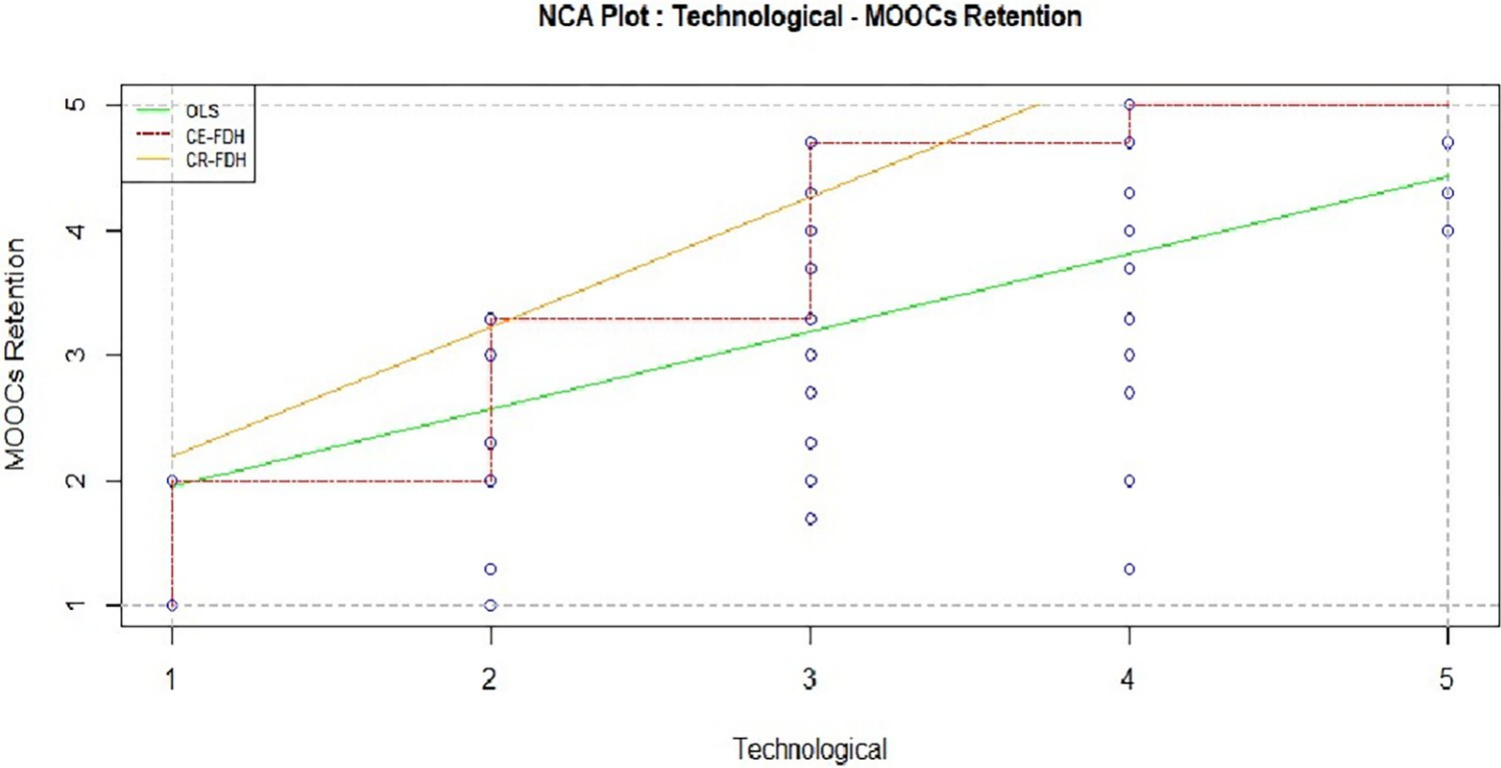
Technological motivation and MOOCs retention.

Statistical findings showed that technological motivation is insignificant (in Table 4, Standardized coefficients: .033) in sufficiency test but have profound effect on necessity analysis (Table 5, Fig 7). Which means technological motivation is the very first important factor to continue the online courses. Without this motivation learners will dropout the course instantly. Only then, MOOCs learners require more than just technological appeal to stay engaged. Factors such as course content relevance, instructional design, and interactive elements play pivotal roles. Learners need content that aligns with their educational goals and a well-structured course that facilitates effective learning. Additionally, collaborative features can enhance the overall learning experience, fostering a sense of community and support. Thus, while technological motivation provides a foundation, a holistic approach incorporating diverse elements ensures sustained retention intention in MOOCs.

#### Quality factors

**Fig 8:**
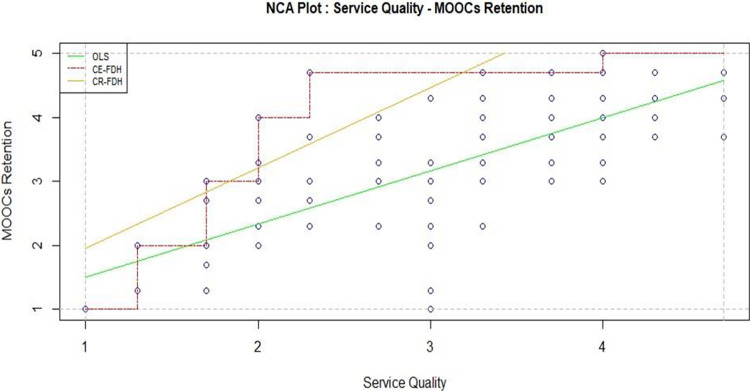
Service quality and MOOCs retention.

The triumvirate of Service Quality, System Quality, and Information Quality constitutes the cornerstone for the sustainable success of Massive Open Online Courses (MOOCs) education. Service Quality, encompassing elements such as responsiveness and empathy, is pivotal in ensuring an enriching learning experience. As MOOCs operate in a virtual environment, effective service delivery becomes synonymous with user satisfaction, fostering a positive perception of the educational platform. Statistical analysis in Table 4 (sufficiency test: standardize coefficient 0.166 with T-value: 4.288) and Table 5 (necessity test: 0.265 with moderate effect) also showed the similar results for this argument that is plotted in Fig 8.

**Fig 9:**
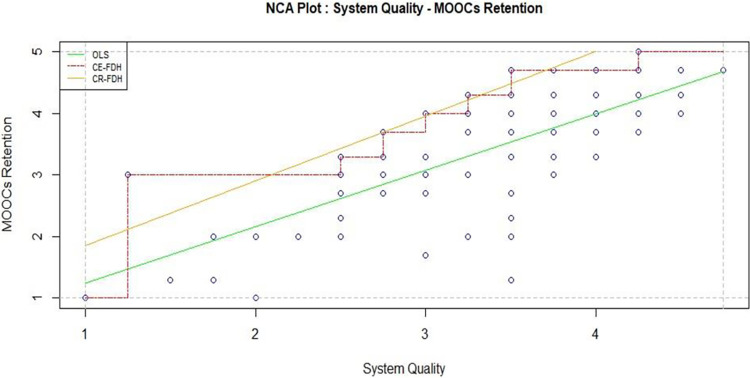
System quality and MOOCs retention.

Simultaneously, a robust and efficient system is imperative for seamless navigation, quick content delivery, and interactive features, all of which significantly contribute to the overall user experience. Inconsistencies or disruptions in system quality could jeopardize the continuity of education delivery. Quantitative results in sufficiency (Table 4: significantly correlate) and necessity (Table 5: large effect also observed in Fig 9) is very much consistent.

**Fig 10:**
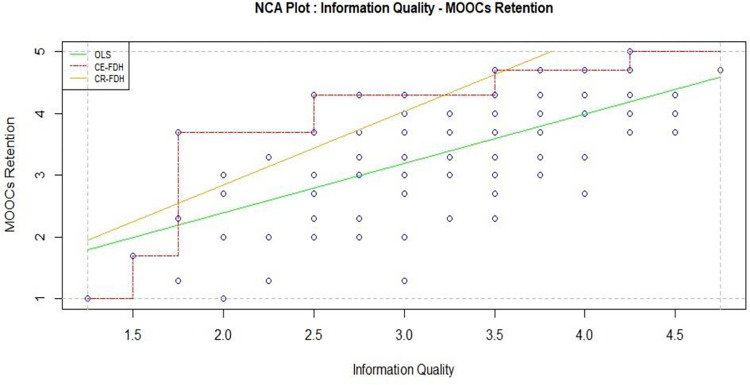
Information quality and MOOCs retention.

Information Quality, the third pillar, high-caliber, up-to-date content not only enhances the learning process but also establishes the credibility of the MOOCs platform. It is very necessary condition to retain in online courses. Results in Table 5 showed large effect (.312) that also found in plotted Fig 10. The findings in Table 4 also consistent with NCA test that showed significant impact (T value: 6.701) on learners’ behavior.

A deficiency in information quality could erode trust and hinder the educational objectives. Therefore, statistical results response in similar tone. In concert, these three dimensions, Service Quality, System Quality, and Information Quality establish a comprehensive framework ensuring not only the initiation but the sustained success of MOOCs education. Their synergistic interplay is not only necessary but sufficient to cultivate an environment conducive to effective and enduring online learning.

## 5. Discussion

Necessary Condition Analysis (NCA) serves as a valuable method for identifying and understanding obligatory conditions contributing to a specific outcome or phenomenon. In the context of MOOCs retention, academic, personal, and technological motivations emerge as necessary success factors crucial for improving retention rates.

Initially, academic motivation is deemed necessary for success in MOOC retention. Learners who lack academic motivation may not actively engage with course materials, complete assignments, or persist throughout the course. Service providers must analyze data to measure the level of academic motivation among learners and pinpoint the juncture at which a deficiency in academic motivation becomes a retention barrier. This analysis may involve examining factors such as quiz participation, assignment completion, and assessment performance.

Secondly, personal motivations, encompassing interest in the subject matter, career goals, and intrinsic motivation, are identified as vital for MOOC retention. Learners are less likely to persist if they do not perceive personal relevance or value in the course. Therefore, course developers should analyze survey responses or other data to discern how learners’ personal motivations correlate with their likelihood of completing the course.

Thirdly, in the context of MOOCs, technological motivations, including ease of use, accessibility, and platform functionality, can significantly impact retention. Technical barriers or negative experiences with the platform may hinder learners’ likelihood of retention.

Fourth, system quality, comprising the technological infrastructure supporting MOOCs, emerges as a necessary condition for retention. NCA underscores the importance of system quality in user engagement and perseverance. Instances of system failures, such as server downtimes or complex user interfaces, demonstrate a statistically significant correlation with dropout rates at various stages of the learning journey.

Fifth, Information quality, referring to the relevance, accuracy, and clarity of educational content, is also deemed a necessary condition for retention. High-quality, comprehensible information enhances learners’ likelihood of persistence, while deficits or ambiguities in instructional content correlate with heightened attrition rates.

Interestingly, social motivation exhibits a comparatively smaller impact on MOOCs retention. While social interactions may enhance the overall learning experience, they may not be a necessary condition for sustained user engagement. Individual variances, as suggested by previous researchers [71], play a crucial role in determining the impact of social motivation, with learners exhibiting diverse responses based on preferences and learning styles.

Finally, course motivation and professional motive, while positively impacting MOOCs retention, have moderate effects. Clear expectations from course motives may lead to higher retention rates, though the impact may not be as pronounced as other factors. Similarly, professional motivation enhances retention but does not represent the sole determinant, with a moderate effect size indicating its impact on MOOCs retention intention.

### 5.1. Theoretical implications

This research contributes significantly to the understanding of MOOCs’ retention intention by highlighting the critical success factors through a necessary condition analysis. The findings underscore the importance of integrating motivational theories and learning engagement frameworks within the context of MOOCs. By identifying key drivers of retention, such as learner satisfaction, perceived usefulness, and course design quality, this study enriches the theoretical discourse on online education. It provides a nuanced understanding of how these factors interrelate and impact learners’ continued engagement, offering a robust theoretical foundation for future research to explore and expand upon these dimensions in various educational contexts.

### 5.2. Practical implications

From a practical standpoint, the insights derived from this study can guide MOOC providers in designing more effective and engaging courses that enhance learner retention. Educators and instructional designers can leverage these findings to optimize course content, delivery methods, and support mechanisms, ensuring they meet the diverse needs of online learners. Additionally, policymakers and educational institutions can use this research to develop strategies and allocate resources towards fostering higher retention rates in MOOCs, ultimately contributing to the democratization of education and lifelong learning. By focusing on the identified success factors, stakeholders can enhance the overall effectiveness and sustainability of MOOCs as a viable educational platform.

## 6. Conclusion

This study suggests that elevating academic, personal, and technological dimensions, coupled with a focus on system and information quality, can serve as crucial strategies for enhancing MOOCs retention. While addressing course motives and professional motivation can contribute positively, their impact is observed to be of a somewhat lesser magnitude. The application of Necessary Condition Analysis (NCA) is instrumental in identifying obligatory factors for MOOCs retention, a task not feasible through regular regression analysis. By isolating necessary conditions, educational institutions and platform providers can strategically target these critical factors to improve overall retention rates. This analytical approach guides the implementation of targeted interventions, such as refining course content, enhancing platform usability, or deploying strategies to boost learner motivation.

In the relentless pursuit of optimizing MOOCs retention rates, the intersection of system quality and information quality emerges as a scholarly essential. NCA provides a methodological lens for discerning these necessary conditions, providing actionable insights for the continual refinement of MOOCs platforms and instructional content. It is captivating to note that social motivation appears to have a comparatively smaller or potentially negligible impact. As scholars and practitioners navigate the landscape of online education, these insights prompt a reevaluation of priorities in MOOCs design, ensuring a nuanced understanding of the role social motivation plays in shaping the learning experience. Aligning instructional strategies with the diverse motivational profiles of learners becomes essential for the field to strive towards a more inclusive and effective online education paradigm.

In conclusion, course motivation and professional motive within the context of MOOCs retention unveil a positive yet moderately influential relationship. Acknowledging the nuanced nature of these motivational dimensions is key for educators, course designers, and policymakers in optimizing MOOCs for enhanced retention. By considering these factors and leveraging them in a balanced manner, the refinement of MOOCs as effective learning tools can be facilitated as they continue to evolve.

## Supporting information

S1 Appendix (DOCX)
