# Supplementary material for: Understanding the success factors of MOOCs’ retention intention: A Necessary Condition Analysis
Source: PLoS One. 2024 Nov 7;19(11):e0310006. doi: 10.1371/journal.pone.0310006 (PMC11542826; doi:10.1371/journal.pone.0310006)
Supplement: S1 Appendix — (DOCX) [file pone.0310006.s001.docx]

**Appendix-A**

| **Variables** | **Items** | **Sources** |
| --- | --- | --- |
| Academic Needs | 1.How important is it for you to earn academic credit directly from professors?  2. What impact do you anticipate this will have on your academic and professional pursuits?  3. What impact do you anticipate this will have on your academic and professional pursuits? | [1, 2] |
| Course Needs | 1)How does the duration of the course align with your availability and commitment levels,  2)Do you find the length suitable for your learning preferences and schedule?  3)Do you believe the level of challenge is appropriate for your learning objectives? | [1, 2] |
| Professional Needs | 1) How confident are you that the successful completion of this course will enhance your professional opportunities?  2) Does the course align with your need for competence and addressing challenges commonly faced in your field of work?  3) What extent do you believe that a course directly related to your job will contribute to your career growth? | [1, 2] |
| Personal Motives | 1) Does your curiosity and personal interest in the course content influence your motivation to enroll?  2) What degree do you believe it aligns with your personal preferences for an enjoyable learning experience?  3) What ways do you anticipate this course contributing to your overall development? | [1, 2] |
| Social Motives | 1) How important is the opportunity to network with others in your field or industry?  2) Considering the value of social support, how does the course facilitate interaction and support from your peers?  3)Does the course contribute to meeting new people and expanding your social circle? | [1, 2] |
| Technological Motives | 1)Does the technological design of this course enhance accessibility for learners?  2)Does the course provide opportunities for interactive design?  3) Do you think this design philosophy will support a comprehensive and holistic understanding of the course content? | [1, 2] |
| Service Quality | 1. How responsive and effective is the customer support provided by the course or platform?  2. Does the system incorporate user feedback to continually improve and enhance the quality of services offered?  3. What measures are in place to provide timely updates and relevant information to users, ensuring a seamless and up-to-date learning experience? | [1, 2] |
| System Quality | 1) What extent do you believe it will meet your expectations for smooth and uninterrupted learning experiences?  2) How user-friendly is the interface of the course platform?  3) The system prioritizes security measures to safeguard personal information and ensure a secure learning environment | [1, 2] |
| Information Quality | 1. Do you believe it aligns with your learning objectives and expectations?  2. Does the system ensure that content is current and timely?  3. Does the system facilitate an effective understanding of complex concepts through its content delivery methods and formats? | [1, 2] |

**References**

1. Bhattacherjee A. Understanding Information Systems Continuance: An Expectation-Confirmation Model. MIS Quarterly. 2001;25(3):351-70.

2. Veeramootoo N, Nunkoo R, Dwivedi YK. What determines success of an e-government service? Validation of an integrative model of e-filing continuance usage. Government Information Quarterly. 2018;35(2):161-74.
